# Frontal evoked γ activity modulates behavioural performance in Autism Spectrum Disorders in a perceptual simultaneity task

**DOI:** 10.1016/j.neulet.2017.11.045

**Published:** 2018-02-05

**Authors:** David A. Menassa, Sven Braeutigam, Anthony Bailey, Christine M. Falter-Wagner

**Affiliations:** a Oxford Human Brain Activity Centre, Department of Psychiatry, OX3 7JX & Wellcome Centre for Integrative Neuroimaging (WIN), Nuffield Department of Clinical Neurosciences, OX3 9DU, University of Oxford, United Kingdom; b Faculty of Medicine, Department of Psychiatry, University of British Columbia, Vancouver, V6T 1Z4, Canada; c Department of Psychiatry & Institute of Medical Psychology, Ludwig-Maximilians-Universität, 80336, Munich, Germany; d Department of Psychology, University of Cologne, 50931, Cologne, Germany

**Keywords:** ADI-R, Autism Diagnostic Interview Revised, ADOS-G, Autism Diagnostic Observation Schedule Generic, ANOVA, analysis of variance, ASD, Autism Spectrum Disorders, DSM-V, diagnostic and statistical manual for mental disorders, fifth edition, MEG, magnetoencephalography, PCA, principal component analysis, TD, typicallydeveloping, WASI, Wechsler Abbreviated Scale of Intelligence, Evoked phase-locked γ-oscillations, Magnetoencephalography, Autism spectrum disorder, Perceptual simultaneity

## Abstract

Autism spectrum disorders (ASDs) are associated with anomalies in time perception. In a perceptual simultaneity task, individuals with ASD demonstrate superior performance compared to typically developing (TD) controls. γ-activity, a robust marker of visual processing, is reportedly altered in ASD in response to a wide variety of tasks and these differences may be related to superior performance in perceptual simultaneity. Using time-frequency analysis, we assessed evoked γ-band phase-locking in magnetoencephalographic recordings of 16 ASD individuals and 17 age-matched TD controls. Individuals judged whether presented visual stimuli were simultaneous or asynchronous. We identified left frontal γ-activity in ASD, which was associated with a reduced perception of simultaneity. Where feature binding was observed at a neurophysiological level in parieto-occipital cortices in ASD in apparent simultaneity (asynchronous stimuli with short delay between them), this did not predict the correct behavioural outcome. These findings suggest distinct γ profiles in ASD associated with the perception of simultaneity.

## Highlights

• Individuals with Autism Spectrum Disorders (ASDs) demonstrate superior performance in a perceptual simultaneity task.
• The properties of the neurophysiological γ-response (30–85 Hz) in this task are not known.
• In 16 ASD individuals, we identify a complex left antero-posterior γ-oscillatory network associated with the perception of simultaneity.
• Frontal γ oscillatory synchrony modulates simultaneity perception in ASD.

## Nomenclature

SIM: No delay condition, ‘*simultaneous*’ response
SHT: Short delay condition, ‘*simultaneous*’ response
LNG: Long delay condition, ‘*simultaneous*’ response

## 1 Introduction

Autism spectrum disorders (ASDs) refer to a range of psychiatric and psychological conditions associated with impairments in social interactions as well as verbal and nonverbal communication. It is commonly agreed, however, that ASD individuals may exhibit unique capabilities, typically observed in the form of superior performance in certain cognitive tasks of visual perception for example [1], [2]. Such superiority has been largely attributed to anomalies in the central coherence of cognitive function [3], [4] although this explanation is not universally agreed upon [5], [6].

Perceptual as well as sensory differences [7] are of particular interest since they are part of the diagnostic criteria for ASD according to the new Diagnostic and Statistical Manual of Mental Disorders, fifth edition (DSM-V). Falling broadly within the category of perceptual differences, a function recently assessed in ASD is the perception of time. Individuals with ASD show a decreased capacity for visual integration over time [8], abnormal perception of time passing [9] and an altered experience of themselves in time [10]. Further studies suggest interval timing abnormalities in ASD [11], [12], [13] whilst some report no differences with respect to the TD population [14], [15]. Mixed results have also been reported by studies on the temporal coding of event structure,^2^ whereby ASD individuals demonstrate lower thresholds for simultaneity [8], [16], [17] and superior performance in the task compared to IQ- and age-matched typicallydeveloping (TD) participants [18].

However, the neurophysiological correlates underlying such superior performance in ASD are not known in perceptual simultaneity. More specifically, oscillatory activity in the γ-range (30–85 Hz), thought to underlie visual processes of perceptual binding and object recognition [19], may play a potential role. In terms of the visual component of the task, decreased evoked γ-activity in visual tasks in ASD has been demonstrated in previous studies [20], [21], [22], [23], [24]. However, very little is known about γ-oscillatory activity in perceptual simultaneity and interval timing and more importantly, whether such activity could underlie performance differences between groups.

We have previously shown that individuals with ASD show superior performance when assessing perceptual simultaneity thresholds [18]. This advantage is associated with differential neural responses and we predict that the neurophysiological response will also be different. We here examine trial-by-trial γ phase consistency to test whether increased γ-oscillatory activity in ASD modulates behavioural performance.

## 2 Methods

### 2.1 Participants

Data from 16 individuals with ASD were analysed. The diagnosis of ASD was confirmed using the Autism Diagnostic Interview Revised (ADI-R) [25] and the Autism Diagnostic Observation Schedule Generic (ADOS-G) [26]. Verbal, performance, and full-scale IQ scores were assessed using the Wechsler Abbreviated Scale of Intelligence (WASI) [27]. Participants with a co-morbid psychiatric disorder, current medication, and a full-scale intellectual quotient (IQ) <85 were excluded. Control data were obtained from 17 TD participants. Written informed consent was obtained from all participants. The ASD and TD groups were matched for chronological age, verbal IQ, and performance IQ and had normal or corrected-to-normal vision. The National Research Ethics Service UK granted ethical approval for the study. Participant details are shown in Table 1. Full details of the sample are reported elsewhere [18].

**Table 1:** Demographic data. Means, and in parentheses, standard deviations and ranges of age, ADOS-G and ADI-R scores of ASD participants as well as the performance, verbal and full-scale IQ measurements of ASD and TD participants are shown (see also [12]).

	ASD (n = 15 M; 1F)	TD (n = 13 M: 4F)
Age in years: months	24:1 (7:0; 16:9–38:3)	26:3 (6:6; 15:9–38:6)
ADOS-G (communication)	3 (2; 1–7)	–
ADOS-G (social interaction)	6 (3; 1–11)	–
ADOS-G (restricted interests)	1 (1; 0–4)	–
ADI-R (social interaction)	17 (5; 10–25)	–
ADI-R (communication)	15 (4; 9–21)	–
ADI-R (restricted interests)	5 (3; 2–10)	–
Verbal IQ	114 (9; 99–127)	114 (12; 99–139)
Performance IQ	112 (15; 75–136)	117 (9; 104–136)
Full-Scale IQ	115 (11; 88–131)	117 (9; 101–141)

### 2.2 Experimental paradigm

A modified version of the perceptual simultaneity task was used [28]. Participants viewed two white bar stimuli, one of which was positioned to the left of the centre of the screen and the other to the right with pseudo-randomized order of bars. We analysed conditions that were presented in pseudo-randomized order: a) the two vertical bars appeared simultaneously on the screen (SIM condition; total display time 17 ms); and b) the two bars appeared asynchronously with either a short 17 ms delay between the two stimuli (SHT condition; both bars disappeared simultaneously 17 ms after onset of the second stimulus) or, as a control condition, a longer 117 ms delay between the two stimuli (LNG condition). Each trial started with a blank screen displayed for 500 ms followed by stimulus presentation. A response cue was presented 1500 ms after stimulus offset. Participants were asked to respond whether stimuli appeared simultaneously or asynchronously by pressing one of two buttons on a keypad. Participants completed four experimental blocks consisting of 60 trials each. Each stimulus condition was presented 80 times for a total of 240 trials. All conditions were analysed for between-group differences. On occasion, for the purpose of presentation and discussion, we refer to the SHT condition as ‘apparent simultaneity’. Further details on the experimental paradigm can be found elsewhere [18].

### 2.3 Data acquisition and data analysis

Measurements were performed using a Neuromag VectorView™ system providing 204 first order gradiometers (data from the magnetometers were not analysed here). Data were sampled at 1000 Hz and corrected for variation in individual head position using MaxMove™. Physiological artefacts were identified. MEG traces with coinciding eye-blinks were corrected using a principal component analysis (PCA)-based approach (in-house software; algorithm based on spatial confound and sensor data correction routines provided by SPM8; see also [29].

### 2.4 Choice of time range

A PCA analysis of the evoked responses across conditions (SIM, SHT, LNG) and two groups (TD, ASD) suggested that 90% (=first component) of evoked power was contained within the 250 ms time period after stimulus presentation. Thus, this time range was chosen for statistical analysis. Note that the actual epoch length was from 100 ms before to 350 ms after stimulus onset in order to include a baseline and to avoid boundary effects due to wavelet calculations.

### 2.5 Choice of the γ-range

The time-frequency analysis was restricted to 30–85 Hz. 30 Hz is commonly regarded as the lower bound of γ-activity. 85 Hz was chosen for the upper limit as it is difficult to establish phase-alignment at higher frequencies because of equipment constraints (notable display of onset jitter).

### 2.6 Time-frequency analysis

γ-band responses were evaluated using a robust measure of phase-locking γ_D_ (γ-density) as described previously [30], [31]. In brief, the method involves a Gabor wavelet transformation and a bootstrap evaluation in order to identify points *(t,f)* in time and frequency of high phase-locking (across stimulus repetition). γ_D_ is defined for each participant, channel and condition. Statistically significant differences between phase-locked *γ*-responses to the simultaneous and asynchronous stimuli were identified as follows. For each channel, a local measure *τ_i_* (applied to γ_D_) of the significance of the difference between phase-locking densities across participants was obtained. Subsequently, a global measure of significance across all channels was obtained:

$$P_{\gamma }\left(t,f\right)≔prob\left(\chi_{\gamma }^{2}\right)=prob\left(-2\overset{N}{\underset{i=1}{\sum }}ln\tau_{i}\left(\gamma_{D}\left(t,f\right)\right)\right)$$

where N denotes the number of channels. *τ_i_* denotes a two-way non-parametric ANOVA (2 groups × 2 conditions) to examine main effects of group, condition and group x condition interaction in *γ* phase-locking. The χ^2^ test is a correction for multiple comparisons because it filters out the effects that are not supported by significant channels. Post-hoc statistical tests included Wilcoxon tests to examine within-group differences in phase-locking and Mann-Whitney *U* tests to examine between-group differences in phase-locking (applied to time-frequency intervals with P**_γ_*(t,f)* *<* *0.01).*Spearman’s correlations were used to examine associations between γ-phase-locking and behavioural responses under various experimental conditions.

## 3 Results

**Fig. 1:**
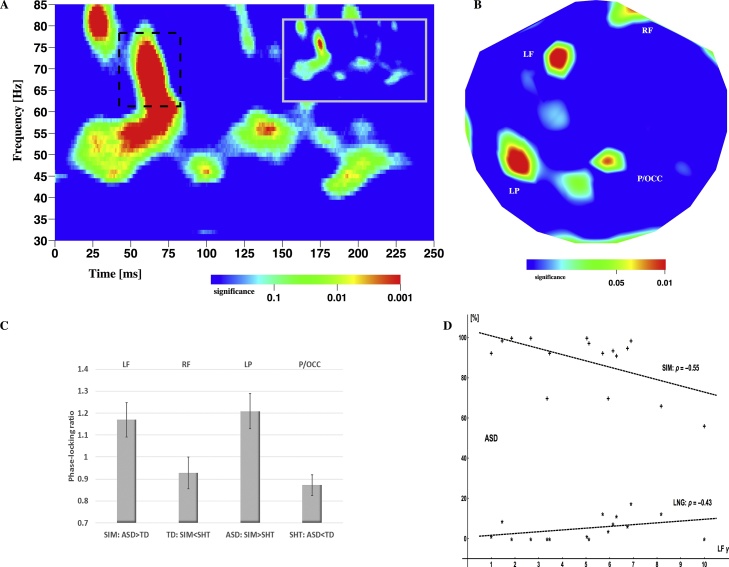
Time-frequency analysis of neuronal responses. A: Significance in the t-f plane obtained from an interaction analysis of two groups (TD, ASD) and two conditions (SIM, SHT). There is a relevant high-γ interaction effect observable at around 62 ms and 70 Hz (dashed box). This effect is also the most significant one in the whole plane (the inset shows the same plane scaled at p = 0.00000001, clearly demonstrating the robustness of the effect). The plane reflects data from all participants and all channels. B: Distribution of significance over the head. There are 4 sites contributing to the global significance shown in A. The insets show the site locations, where a template brain has been superimposed for visualisation (RF: right frontal, LF: left frontal, LP: left parietal/posterior and P/OCC: parieto-occipital). C: Post-hoc analysis. Each site exhibits one significant comparison illustrated as a phase-locking ratio (see text). For example, channel LF is associated with a significant increase in phase locking in ASD compared to TD participants in condition SIM. D: Behavioural-Neural correlations. High γ phase-locking and ‘simultaneous’ responses correlate in both the SIM and LNG conditions in ASD participants, but not in TD subjects. For graphical presentation, linear regression lines are shown, where the γ-values have been transformed to the interval [1], [10].

The two-way non-parametric ANOVA showed a complex pattern of significant interaction effects in the time-frequency plane 0–250 ms after stimulus onset and at 30–85 Hz in frequency (Fig. 1A). Using the main factor (group, condition) effects and the results from separate analyses (within ASD and within TD, median splitting groups according to the number of simultaneous responses in the SIM condition), the pattern could be narrowed down to a single cluster of significance located around 62 ms and 70 Hz (dashed rectangle). The cluster was located to the high γ-band range at latencies well before main activation peaks were seen in the evoked responses. It spans 36 ms in time and 18 Hz in frequency (uncertainties inherent to the wavelet approach).

**Fig. 2:**
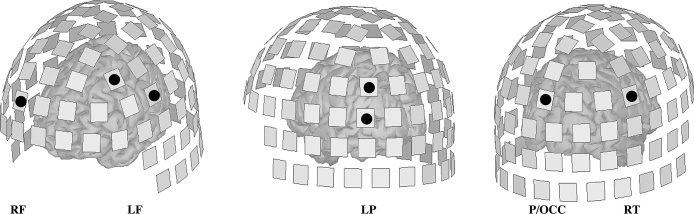
Location of significant γ-band effects in sensor-space. A template brain has been superimposed for visualisation (based on average head-to-helmet position across subjects). A total of 7 channels account for the two γ effects shown in 3 . The channels are over the right frontal (RF), left frontal (LF), left parietal/posterior (LP), parieto-occipital (P/OCC) and right temporal (RT) areas.

This cluster of (global) significance corresponded locally to four significant channels over the head (Fig. 1B). A post-hoc analysis revealed that each of these sites showed one significant difference (out of six comparisons; Fig. 1C). In ASD participants significantly higher phase-locked activity in the γ-band was evoked compared to TD participants in the SIM condition over left frontal areas. Notably, this γ activity in individuals with ASD was negatively correlated with both the number of SIM responses (Spearman ρ = −0.55, p < 0.01; Fig. 1D) and, to a lesser degree, the number of SHT responses (ρ = −0.39, p < 0.05). Moreover, left frontal γ activity was positively correlated with the number of LNG responses (ρ = 0.43, p < 0.01; Fig. 1D). No such correlations were present in TD subjects. The approximate locations of this site and other significant sites over the brain are shown in Fig. 2.

In contrast, TD participants evoked significantly higher phase-locked activity compared to individuals with ASD in the SHT condition over parieto-occipital areas.

The two other sites showed within-group differences. In TD participants, phase-locked γ-responses were stronger in the SIM condition compared with SHT over right frontal areas. In individuals with ASD, phase-locked γ-responses were stronger in the SIM condition compared with SHT over left posterior parietal areas.

**Fig. 3:**
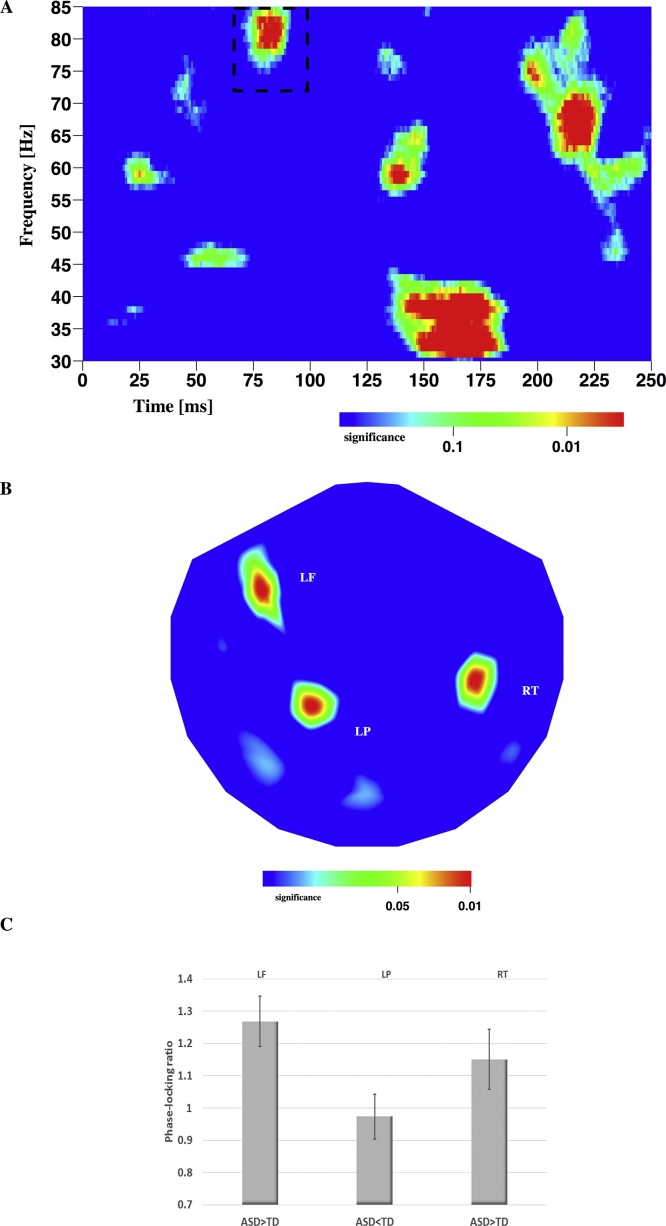
Time-frequency analysis of neuronal responses in the long delay condition. A: Global significance obtained from a between-group comparison of responses. A relevant high-γ effect is observable at around 81 ms and 80 Hz (dashed box). Note that several effects throughout the γ-range are observable after onset of the second bar (115 ms). The plane reflects data from all subjects and all channels. B: Distribution of significance over the head. There are 3 sites contributing to the global significance shown in A. C: Post-hoc analysis. The 3 comparisons are illustrated as a phase-locking ratio. Note that the effect in LP is weak (p < 0.1).

The results obtained from a non-parametric, between-group comparison of the responses in the LNG condition also showed a complex pattern of significant interaction effects in the time-frequency plane (Fig. 3A). The majority of the effects were observed after the second bar appeared and were not considered further. Following the presentation of the first bar, a strong effect in the high γ-band was identified at 81 ms and 80 Hz, spanning 32 ms in time and 20 Hz in frequency. Note that this cluster significantly overlaps in both time and frequency with the cluster of significance identified in the interaction analysis.

This cluster of significance corresponded locally to three significant channels over the head (Fig. 3B). ASD participants evoked significantly higher phase-locked γ-band responses compared to TD participants over left frontal areas (Fig. 3C). Note that this site was in close proximity to the left frontal area identified above. Activity observed over left parietal regions was nearly identical in the groups (less in ASD as a trend). Finally, ASD participants evoked significantly higher phase-locked γ-band responses compared to TD participants over right temporal areas.

## 4 Discussion

The current study investigated oscillatory activity in the γ-range in ASD in a perceptual simultaneity task. This investigation was prompted by earlier findings of participants in ASD suggesting that individuals exhibited an enhanced visual resolution compared to TD when presented with stimuli separated by short time intervals in the millisecond range compared to simultaneously presented stimuli and stimuli separated by relatively long intervals [16], [18]. The current findings suggest that such condition-specific sensitivity is, at least to some extent, facilitated by activity in a complex fronto-parietal fast network commonly associated with higher order processes.

Accordingly, the increased left frontal γ activity might indicate a process driving ASD individuals away from judging stimuli to be simultaneous in the SIM condition, while the same activity might facilitate superior performance in the SHT condition, although the latter interpretation is only indirectly supported in these data. Concomitantly, in the SHT condition, decreased parietal-occipital γ phase-locking may be suggestive of perceptual discrimination in ASD consistent with perceptual feature un-binding [32], i.e. a mechanism driving ASD individuals away from perceiving simultaneity, thereby increasing performance in the SHT condition. In these data, parieto-occipital γ in ASD participants correlates with behaviour only at the group level (i.e., is lower in ASD compared to TD, where ASD have a higher rate of non-simultaneous responses). These findings are consistent with intact bottom-up processing in parieto-occipital regions in ASD as opposed to altered top-down processing explaining increased left frontal activity in this group. Neuronal activity in other brain areas might further facilitate this increased sensitivity in ASD beyond and possibly independently of this putative fronto-parietal network, as suggested by increased γ phase-locking following SIM compared to SHT stimuli over left parietal cortices. The precise significance of this effect is unresolved but it is conceivable that retrieval of episodic memory may play a role here [33].

At present, it is not clear if the γ-activities observed over left frontal cortices have the same or similar functional significance in LNG condition in ASD. Speculatively, an excess of γ-activity can be beneficial for short interval detection but may heighten error rates for longer temporal separation of stimuli. This view emphasizes that the notion that perceptual superiority in ASD is not universal but dependent on the task condition at hand.

Frontal activity and aberrant fronto-parietal networks have been described in ASD [34], [35], [36]. Note that in our study, this does not exclude the possibility that ASD individuals might achieve higher performance in the SHT condition by relying to a greater extent on low-level processing compared to TD participants as previously conjectured. Conceptually, the findings are broadly in line with heightened sensory and perceptual discrimination in ASD in the visual domain [37]. The enhanced temporal resolution in the SHT condition is broadly in line with the theory of weak central coherence [3] and an extension thereof from the spatial to the temporal domain (see [16]). Accordingly, a detail-focused approach to parts of stimuli might explain why individuals with ASD perform better in the near-threshold condition.

Interestingly, early latency γ-activity is also modulated by the task in TD controls. Speculatively, the increased activity to SHT compared to SIM stimuli observed over right frontal cortices is indicative of some form of decision-making aimed at counteracting the ‘primitive’ reflex of perceiving the two bars simultaneously, although this process is not reflected in the behavioural response [38]. Irrespective of the precise meaning, the γ effects observed here suggest that TD individuals and participants with ASD employ partially different neuronal pathways in order to solve the task.

As a limitation, it was not possible to investigate data associated with “non-simultaneous” judgments in the SHT condition or with “simultaneous” judgments in the control condition with a longer delay because of much fewer epochs available for comparison. We suggest future MEG studies to employ a range of intermediary conditions, as we had previously used in the complete psychophysical task version [16]. Further work is also warranted to investigate the lower α and β frequency rhythms [39] coupled with γ-oscillatory dynamics to further unravel the neural correlates of time perception and their relationship to task performance in ASD. A lack of complete magnetic resonance imaging scans of all participants restricted analysis to signal space. Arguably, acquiring structural scans from all participants with ASD in a sample will remain to be a challenge for future research and is likely to lead to unwanted and uncontrollable self-selection of study participants.

## 5 Conclusion

This study examined γ-phase-locking underlying altered performance in perceptual simultaneity in ASD. Left frontal and parietal γ phase-locking may be part of a network specifically affecting the coding of temporal event structure in ASD. With ASD being termed a ‘dis-connectivity syndrome’ with impairments in local and long-range synchronization in the γ-band [40], the discovery of characteristic neural patterns may provide potential biomarkers for these disorders and extend our understanding of the clinical phenotype.

## Funding

This work was supported by the Oxford Human Brain Activity Centre & Department of Psychiatry, a project grant from the Bailey-Thomas trust held by Professor Anthony Bailey (award number: 2216/1) and a Clarendon scholarship at the University of Oxford to Dr. David A. Menassa.

## Competing interests

The authors declare that they have no competing interests.

## Ethics approval and consent to participate

The National Research Ethics Service UK granted ethical approval for the study and all participants consented to participate.

## Footnotes

2 Perceptual simultaneity tasks measure functioning of the coding of temporal event structure, whereby participants have to judge whether the simultaneity or asynchrony of an event and their relative order. Simultaneity thresholds here are operationalized as the temporal delay between two visual events above which participants can tell that the events happened asynchronously and below which events are perceived as simultaneous.
